# On Topological Analysis of fs-LIMS Data. Implications for in Situ Planetary Mass Spectrometry

**DOI:** 10.3389/frai.2021.668163

**Published:** 2021-08-23

**Authors:** Rustam A. Lukmanov, Andreas Riedo, David Wacey, Niels F. W. Ligterink, Valentine Grimaudo, Marek Tulej, Coenraad de Koning, Anna Neubeck, Peter Wurz

**Affiliations:** ^1^ Space Research and Planetary Sciences (WP), University of Bern, Bern, Switzerland; ^2^ Centre for Microscopy, Characterisation and Analysis, The University of Western Australia, Perth, WA, Australia; ^3^ Department of Earth Sciences, Uppsala University, Uppsala, Sweden

**Keywords:** fs-LIMS, mass-spectrometry, UMAP (uniform manifold approximation and projection), mapper, microfossils, mars, Gunflint

## Abstract

In this contribution, we present results of non-linear dimensionality reduction and classification of the fs laser ablation ionization mass spectrometry (LIMS) imaging dataset acquired from the Precambrian Gunflint chert (1.88 Ga) using a miniature time-of-flight mass spectrometer developed for *in situ* space applications. We discuss the data generation, processing, and analysis pipeline for the classification of the recorded fs-LIMS mass spectra. Further, we define topological biosignatures identified for Precambrian Gunflint microfossils by projecting the recorded fs-LIMS intensity space into low dimensions. Two distinct subtypes of microfossil-related spectra, a layer of organic contamination and inorganic quartz matrix were identified using the fs-LIMS data. The topological analysis applied to the fs-LIMS data allows to gain additional knowledge from large datasets, formulate hypotheses and quickly generate insights from spectral data. Our contribution illustrates the utility of applying spatially resolved mass spectrometry in combination with topology-based analytics in detecting signatures of early (primitive) life. Our results indicate that fs-LIMS, in combination with topological methods, provides a powerful analytical framework and could be applied to the study of other complex mineralogical samples.

## Introduction

The current state of space exploration is on the verge of new frontiers, holding promise for discoveries on other planetary bodies through *in-situ* robotic exploration (Vago et al., 2015). For example, Mars and the icy moons of Jupiter and Saturn, once thought to be lifeless, have gained more attention from the scientific community in recent decades due to new data informing upon the potential habitability of these bodies (Priscu and Hand 2012; Garcia-Lopez and Cid 2017; McMahon et al., 2018). Thus, there is an ongoing need for sensitive and high-throughput space instrumentation providing precise analytical data on a microscale (Navarro-González et al., 2006; Goesmann et al., 2017). However, space-type instruments are usually small and provide only a fraction of the sensitivity and overall capability of their full-scale laboratory counterparts. Reduction in performance occurs due to the strict constraints on size, power consumption, and weight of the scientific payload. Therefore, the development of new miniature instruments and analytical methods with improved capabilities is a continuously pressing issue (Li et al., 2017; Arevalo et al., 2018; Stevens et al., 2019; Wurz et al., 2020).

Laser-based mass spectrometry (Laser Ablation Ionization and Desorption Mass Spectrometry–LIMS and LDMS) is a modern and compact analytical method that promises to greatly enhance the quality of chemical analysis on planetary bodies (Riedo et al., 2013b; Arevalo et al., 2018). The first LIMS instrument selected and built for a planetary lander was LASMA, developed for the Phobos-Grunt mission (Managadze et al., 2010). Recently, the second LIMS instrument was chosen for the upcoming ExoMars mission/Rosalind Franklin Rover (Goesmann et al., 2017), further facilitating developments in this field. Laser-based mass spectrometry, developed for *in-situ* planetary exploration, as a versatile method, can provide a description of molecular composition and element, isotope characterization of solids (Moreno-García et al., 2016; Arevalo et al., 2020; Tulej et al., 2020). The time-of-flight version of LDMS has been shown to be capable of measuring extremely low concentrations (fmole) of amino acids in the desorption mode (Ligterink et al., 2020). LIMS modification of this instrument has been reported to measure ppbw level trace elements and routinely measure fine chemistry from a variety of samples (Riedo et al., 2013a; Neuland et al., 2016; Wiesendanger et al., 2017). Moreover, a number of reports have indicated that LIMS, particularly fs-LIMS, might be applicable to the detection of faint signatures of life from microscopic inclusions (Tulej et al., 2015; Wiesendanger et al., 2018) and low-biomass Martian analogs (Stevens et al., 2019; Riedo et al., 2020). However, the field of study of early and primitive life remains profoundly complex (Brasier and Wacey 2012; Westall et al., 2015; Wacey et al., 2016) with no single chemical criterion that can be assigned as definitive proof of biogenicity. A number of authors have proposed a multi-criteria approach, where a multitude of methods needs to be applied before any conclusions can be drawn (Hofmann 2008; Brasier and Wacey 2012; Hand et al., 2017; Vago et al., 2017; Neveu et al., 2018; Chan et al., 2019). The multi-method approach enhances the size of parametric space and reduces the probability of false-positive detection. Therefore, any advancement within each of the applied methods can increase the overall confidence of the correct identification of signatures of life.

In this contribution, we hypothesize that on the basis of the full feature scale (mass range) present in the fs-LIMS spectral datasets, it is possible to identify minerals and compounds of specific chemistry using an unsupervised data-driven approach. We describe a topology-based analysis pipeline to define the complexity of the fs-LIMS imaging data in low dimensions and identify groups of spectra that share a significant degree of similarity. We apply the aforementioned method to 18,000 composite spectra acquired from the Gunflint chert (1.88 Ga), which contains populations of well-preserved Precambrian microfossils of proven biological origin (Wacey et al., 2013). The analysis of the data reveals four distinct populations of fs-LIMS spectra, which correspond to two groups of microfossils, the quartz matrix in which microfossils are entombed and organic surface contamination spectra. Moreover, we describe a fine transitional structure between classes and argue that low dimensional representations are of high utility in *in-situ* mass-spectrometry and space research. Further, we speculate that our approach is scalable to non-space instruments and may, therefore, prove useful in the field of Precambrian micropaleontology and high-dimensional analytical chemistry in general.

## Methods

In this study, we use laser ablation ionization mass spectrometry for the characterization of the chemical composition of the Gunflint sample and optical microscopy to identify morphological features. A thorough review of LIMS operation principles, current state-of-the-art, and application case studies can be found in a number of previous reports (Tulej et al., 2014; Wiesendanger et al., 2017; Grimaudo et al., 2020; Ligterink et al., 2020) and reviews (Grimaudo et al., 2019; Azov et al., 2020), and therefore, only a short description will be given here. In the simplest case, LIMS instruments include two main parts–a pulsed laser system to ablate and ionize materials and a mass analyzer to separate and register ions produced during the ablation and ionization process. The fs-LIMS is a successor of ns-LIMS, with the only difference that the mass analyzer is coupled to the fs laser system. Current commercial fs lasers can provide peak power fluences up to terawatt/cm^2^, compressed to very short pulses of femto-second duration. Such high powers can ionize any material, thus, providing means for an isotope and element characterization of any solid with very small detection limits and reduced matrix effects (Riedo et al., 2013c). As an ion source, we have installed a Ti:Sapphire laser with chirped pulse amplification, which provides a stable IR-775 nm, ∼190 fs laser radiation. Conversion of the fundamental wavelength from IR-775 nm to UV-258 nm was made using a commercially available third-harmonic generation module.

**FIGURE 1:**
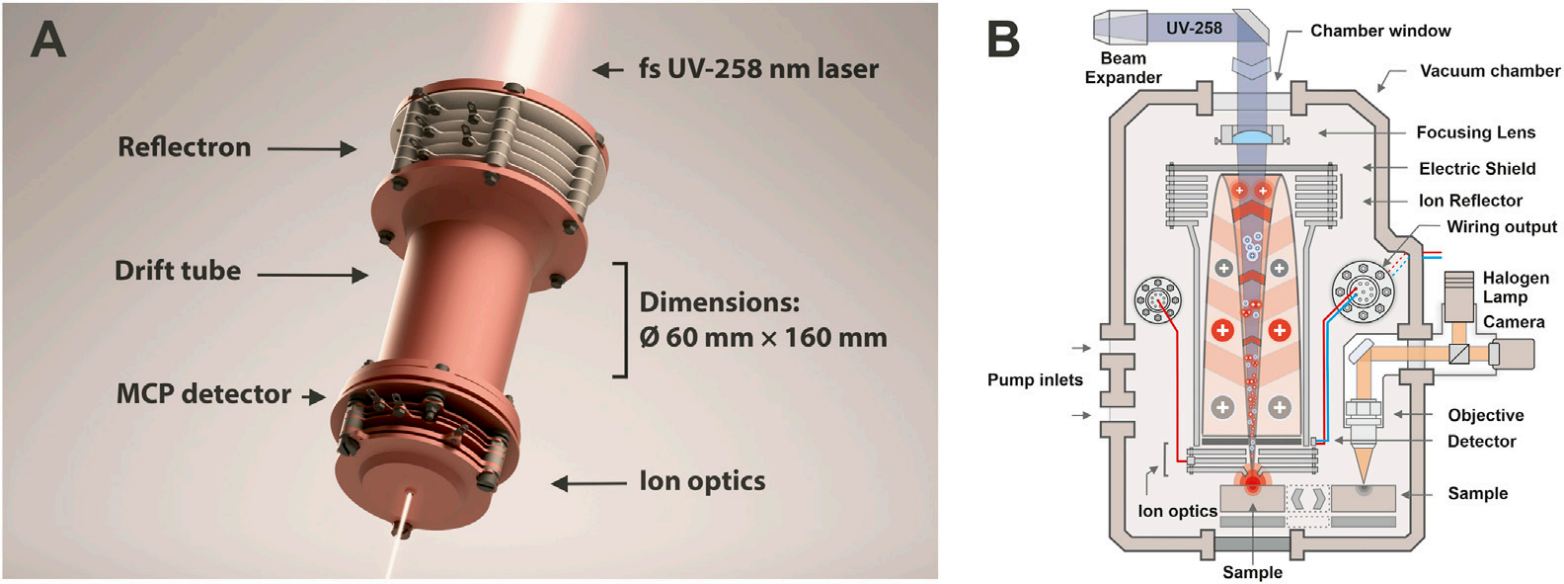
**(A)** 3D render of our miniature time-of-flight mass analyzer. Location of the reflectron, drift tube, entrance ion optics, MCP detector, and dimensions of the instrument are denoted. The focusing fs-UV laser light shown on the top and the bottom and illustrates an axial design of the mass analyzer. Sample positioning is not shown. However, in the laboratory setting, the investigated sample is positioned in close vicinity to the entrance plate of the ion optical system of the mass analyzer, right in the position of the laser focus, to achieve ablation and subsequent ionization of target material. **(B)** Schematic illustration of the fs-LIMS. An fs-laser radiation (blue line) ablates and ionizes material from the sample. The positively charged ions are separated and detected using the time-of-flight mass spectrometer. The ablation position can be precisely located using an integrated microscopy system.

The fs-LIMS system used in this study consists of a miniature time-of-flight (TOF) mass analyzer (⌀ 60 × 160 mm) (*see* Figure 1) with an axially symmetric design and single unit mass resolution. The instrument was developed for *in-situ* space applications, and due to its miniature design it could be placed on a rover, lander, or even used as a handheld instrument (Wurz et al., 2020). In normal operation mode, fs-LIMS could identify major chemical composition along with ppmw-level concentrations of trace elements. As shown in Figure 1, the TOF mass analyzer consists of entrance ion optics (where ions are confined and accelerated), a drift tube (where ions experience mass/charge separation), a reflectron (ion mirror which uses an electric field), and a microchannel plate (MCP) detector system (Riedo et al., 2017) to register ion flux. The schematic illustration of the fs-LIMS sample analysis is shown in Figure 1B. The focused blue light indicates the fs-UV-258 nm laser radiation that passes through the instrument and ablates the small area of the sample with a diameter of the ablation spot of about 5 µm. The positioning of the ablation spots is determined by the internal microscopy system. The objective of the microscope is located at a fixed offset from the instrument. After ablation and ionization, positively charged ions are guided by an electric field of the instrument into a defined parabolic trajectory so that every ion that enters the instrument will land on the surface of the detector. Incoming time-separated ion flux launches an electron avalanche within the microchannels of the detector system and creates a measurable current on the output anodes. Thus, time-of-flight LIMS measures an output current per unit of time, which is correlated with the element and isotope abundances of an investigated spot. Note that the image of the fs-laser beam passing through the instrument (Figure 1A) is exaggerated - in the laboratory setting, the laser focal point is located in close proximity to the entrance electrode of the mass analyzer (*see* Figure 1B).

**FIGURE 2:**
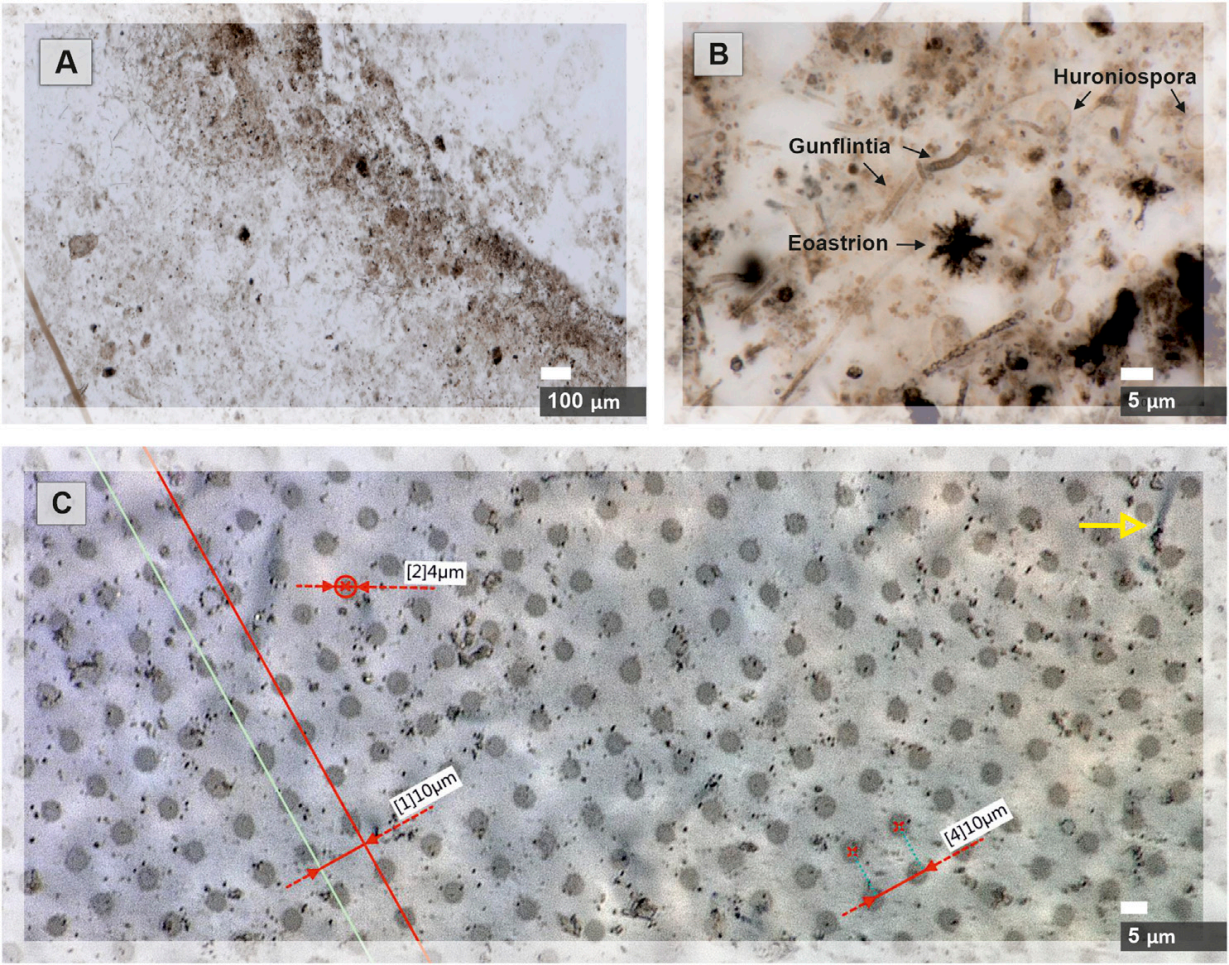
Microscope images of the Gunflint chert before and after the fs-LIMS imaging campaign are shown. **(A)** Microscope image of the area (0.9*2 mm^2^) chosen for the chemical imaging with our fs-LIMS system. The dark brown patches distributed through the sample and forming a diffuse layer in the middle of the picture represent a bio-lamination surface. **(B)** Close-up microscope picture of individual microfossils from the bio-lamination surface. Filamentous (Gunflintia), star-shaped (Eoastrion), and spherular microfossils (Huroniospora) can be seen. **(C)** Microscope picture of laser ablation craters (0.9*2 mm^2^ area covered with 90*200 pixels–18,000 ablation positions) formed after the fs-LIMS imaging campaign. Red lines denote the accuracy of sample positioning (the gap between ablation craters is consistently 10 µm) and identify the ablation crater diameters. Individual craters range in diameter from 4 to 5 µm. Note, on the upper part of the image, the yellow arrow indicates an individual microfossil body. The size of the microfossil can be compared with the diameter of the analytical spot.

The investigation of a 30 µm thick thin-section of Gunflint chert has been conducted with our miniature fs-LIMS system. The sample acquired from the Gunflint Formation (Schreiber beach locality, Ontario, Canada; Wacey et al., 2012, 2013) represents a finely polished thin slice of the original rock, glued to the glass substrate and mounted on a steel holder. Preliminary optical microscopy was performed on the sample to identify specific areas of microfossil aggregation (*see* Figure 2A). Matrix material in which microfossils are preserved was identified to be microcrystalline quartz. Chemical imaging of the rectangular area, containing a bio-lamination surface (aggregation of microfossils within a stromatolite) and a clear host area (quartz filled matrix) was done with the LIMS system using the fs UV-258 nm laser, which provides a flux of 4.8 eV UV photons, which is well suited for ionization of glasses and other non-conductive materials with low absorption coefficients.

Figure 1A. 3D rendering of our miniature time-of-flight mass analyzer. Location of the reflectron, drift tube, entrance ion optics, MCP detector, and dimensions of the instrument are denoted. The focusing fs-UV laser light shown on the top and the bottom and illustrates an axial design of the mass analyzer. Sample positioning is not shown. However, in the laboratory setting, the investigated sample is positioned in close vicinity to the entrance plate of the ion optical system of the mass analyzer, right in the position of the laser focus, to achieve ablation and subsequent ionization of target material. Figure 1B. Schematic illustration of the fs-LIMS. The fs-laser radiation (blue line) ablates and ionizes material from the sample. The positively charged ions are separated and detected using the time-of-flight mass spectrometer. The ablation position can be precisely located using the integrated microscopy system.

### Data Acquisition

**FIGURE 3:**
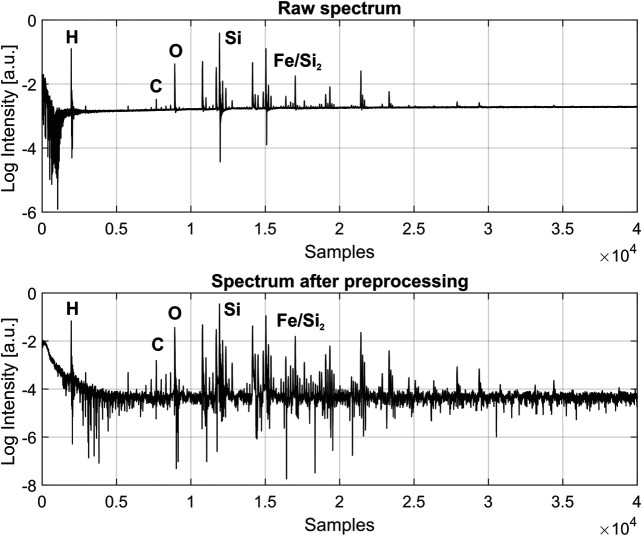
Comparison of fs-LIMS spectra (composite spectrum of 200 laser shots, recorded from single position), before and after data preprocessing, acquired from the Gunflint chert sample. Each line in the spectrum represents a single unit mass. The increase of SNR to 10^4^ and correction of the baseline can be noted. *See* the text for the full description of preprocessing procedures. Exemplary atomic lines are denoted on top of the spectrum.

A rectangular area of 0.9 × 2 mm^2^ was investigated using the fs-LIMS system (*see* Figure 2A). A relatively low number of laser pulses were applied to each surface position – 200 laser shots, to avoid material displacement and crater-to-crater cross-contamination. The spatially resolved measurements conducted on the Gunflint chert resulted in the collection of 18,000 composite spectra (collected from the grid - 90 by 200 position or ablation sites). A composite spectrum collected from the given position (or ablation site) resulted in the accumulation of 200 single-shot spectra, with 64,000 data points digitized per spectrum. Thus, the total number of registered shots resulted in 3.6 ×·10^6^ single-shot spectra. The laser energies applied to each position amounted to ∼360 nJ/pulse (measured at the sample surface) using UV-258 nm laser. This energy was appropriate to produce the optimal quality for the mass-spectrometric signal, both from the microfossils and the quartz-filled host area. Analytical conditions were held constant during the data collection. The diameter of the average ablation crater was measured to be ∼5 μm, and gaps between ablation craters were set to 10 µm (*see* Figure 2C). A custom-built software package was used to control the translation stage and the laser firing intervals. A fast data acquisition system from Keysight was used for digitizing current from the anodes, providing a 3.2 GSa/s sampling rate. An example of a single composite spectrum (representing a histogram of 200 individual single-shot spectra) registered from the Gunflint sample is shown in Figure 3. A single mass spectrum consists of 64,000 individual datapoints sampled with a digitizer, where each digitized data point corresponds to ∼0.33 ns of a flight time. Thus, every recorded spectrum contains information about ∼20 µs of a flight time, which provides a mass/charge (m/z) coverage of up to 800 m/z, providing a complete record of all stable isotopes and simple molecular compounds. Overall, 18,000 composite spectra were collected from the Gunflint sample, with a 10 µm gap between ablation craters. Additionally to the mass spectra collection, noise measurements were recorded, which allowed the enhancement of the recorded signal.

Figure 2. Microscope images of the Gunflint chert before and after the fs-LIMS imaging campaign are shown. A) Microscope image of the area (0.9*2 mm^2^) chosen for the chemical imaging with our fs-LIMS system. A. The dark brown patches distributed through the sample and forming a diffuse layer in the middle of the picture represent a bio-lamination surface. B) Close-up microscope picture of individual microfossils from the bio-lamination surface. Filamentous (Gunflintia), star-shaped (Eoastrion), and spherular microfossils (Huroniospora) can be seen. C) Microscope picture of laser ablation craters (0.9*2 mm^2^ area covered with 90*200 positions – 18,000 ablation sites) formed after the fs-LIMS imaging campaign. Red lines denote the accuracy of sample positioning (the gap between ablation craters is consistently 10 µm) and identify the ablation crater diameters. Individual craters range in diameter from 4 to 5 µm. Note, on the upper part of the image, the yellow arrow indicates an individual microfossil body. The size of the microfossil can be compared with the diameter of the analytical spot.

### Data Preprocessing

The entire imaging dataset, which consists of ∼50 GB of recorded composite mass spectra, was preprocessed before any analysis was applied to the data. A mass spectrometry preprocessing routine applied to the dataset consists of several typical steps that largely follow methods described in (Gil and Marco 2007) and (Meyer et al., 2017). The fs-LIMS preprocessing routine applied to the imaging data consisted of:

1) Noise removal for an improvement of the signal-to-noise ratio (SNR) of the signal. The noise signal (empty composite mass spectra) was recorded after the imaging campaign was completed. The recorded noise waveform was subtracted from the imaging observations.
2) Baseline subtraction. A filter function was applied to the noise-removed mass spectra to estimate varying baseline within multiple windows and regressed using spline approximation.
3) Jitter correction. Since materials within the analyzed sample might be of better or worse ionization efficiency (mainly due to topography), temporal variation of ion yields is expected to occur so that times-of-flight of given ion packets might slightly vary. Typically, this effect is small and affects the peak shapes in a minor way. However, since we collected a relatively large dataset, a correction procedure has been applied. To correct for mismatch of times-of-flight, we have used an autocorrelation function described in (Gil and Marco 2007).
4) Low pass filtering. The low pass filter with normalized cutoff frequency at 0.13 πrad/sample and stopband attenuation of 60 dB was applied to each composite mass spectrum. This step removes the remaining high-frequency component from the recorded signal. Typically, it improves the SNR by two to five and does not alter the peak shapes.
5) Parametric peak preserving smoothing. The Savitzky–Golay filter function (Press and Teukolsky 1990) was applied to flatten the baseline further and increase the SNR.
6) Mass scale assignment. An average time-of-flight spectrum of all 18,000 spectra was recalculated for mass calibration purposes. A simple quadratic equation was used to calibrate the mass scale with the time-of-flight spectrum (Riedo et al., 2013a).
7) Single mass unit decomposition. An integration of consecutive 260 single unit masses, starting from ^1^H, was achieved by recalculating the time-of-flight windows from the mass calibration equation and utilizing direct Simpson’s integration (Meyer et al., 2017).

**FIGURE 4:**
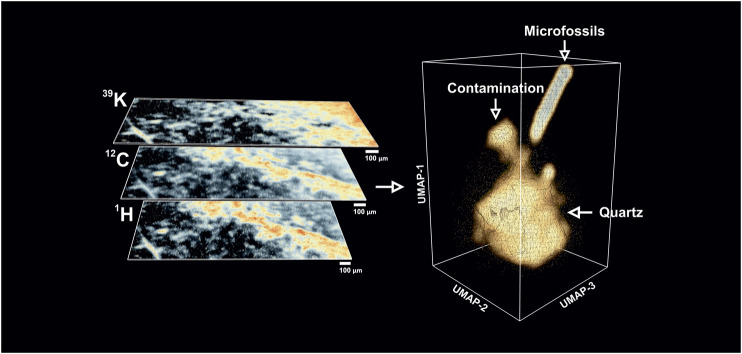
Left panel–Exemplary isotope intensity maps (warmer colors indicate high concentrations) retrieved from the fs-LIMS mass spectra. The bio-lamination surface (aggregation of microfossils) could be identified from ^12^C and ^1^H maps (bright yellow to red areas), distribution of ^39^K indicates the presence of the surface contamination (upper left corner, bright yellow to the red area). Dark areas on the isotope maps indicate low-intensity regions and correspond to the quartz matrix. To compare with an optical image, *see* Figure 2A. Right panel–Low dimensional structure of the imaging data cube revealed by UMAP. Triangulated mesh represents volumetric isodensity surface of UMAP scores calculated from the 18,000 fs-LIMS mass spectra. Three separate entities could be observed from the spectral neighborhood, namely quartz, contamination, and microfossils. The point cloud data plotted along with the density surface.

Figure 3 shows a typical raw spectrum (top panel) acquired from the Gunflint sample before any data preprocessing has been applied. The bottom panel shows a spectrum after preprocessing and reveals significantly improved SNR (10^4^) and a flat baseline. After step number seven, multiple isotope maps were calculated using Kriging interpolation (further information in the text and *see* Figure 4) for an investigation of the distribution of major abundant elements. The imaging dataset was z-score normalized to remove the imbalanced scales. An assessment of the pairwise correlation factors was made, showing that approximately half of the dimensions (single unit masses) are empty or very weakly expressed.

The principal component analysis (PCA) reduction down to the first 60 principal components was applied to remove empty dimensions dominated by noise from the original dataset. The Uniform Manifold Approximation and Projection (UMAP) algorithm (McInnes et al., 2018) was used to further characterize non-linear dependencies present in the PCA reduced data matrix. The overall classification of the UMAP scores was made using a hierarchical density-based clustering algorithm (HDBSCAN) (Campello et al., 2013; McInnes et al., 2017). The specific spectra identified from the microfossils were further visualized using the Mapper algorithm (Singh et al., 2007). The identification of the modules present in the Mapper network was conducted using a greedy modularity optimization algorithm (Louvain) (Blondel et al., 2008).

Figure 3. Comparison of fs-LIMS spectra (composite spectrum - 200 laser shots, recorded from single pixel), before and after data preprocessing, acquired from the Gunflint chert sample. Each line in the spectrum represents a single unit mass. The increase of SNR to 10^4^ and correction of the baseline can be noted. *See* the text for the full description of preprocessing procedures. Exemplary atomic lines are denoted on top of the spectrum.

## Results

We calculated the intensity maps of major (abundant) isotopes to understand a basic representation of the data. In Figure 4, the spatial distributions of ^12^C, ^1^H, and ^39^K are illustrated and the chemical maps reveal specific areas where isotopes show elevated intensities. In comparison with the optical image of the same area (*see* Figure 2A), one can see that most of the dark brown patches identified from optical microscopy as microfossils preserved in the bio-lamination surface are spatially correlated with increased values of ^12^C and ^1^H. This observation is consistent with the fact that major elements within microfossil bodies are C and H. However, the intensity map of ^39^K reveals different distribution. A top-right corner of the sample, which was previously identified as a clean matrix (milky quartz), reveals elevated concentrations of ^39^K and relatively intense ion yields of ^12^C. In fact, after a closer investigation of the mass spectra recorded from that region, we identified a full range of biorelevant elements (CHNOPS).

Additionally, a full range of Si isotopes, various silicon oxides, and small chains of hydrocarbon clusters were observed from that region. Considering that a particular location from optical microscopy does not show any distinct mineralogical association with described elements, we concluded that the identified area could belong to the organic contamination. From our previous studies of the Gunflint sample (Wiesendanger et al., 2018), particularly the chemical depth profiling of the neighboring region, it was identified that organic contamination is present as a thin surface layer and organic spectral features quickly decay with increasing depth. The organic contamination potentially comes from the sample handling and preparation procedures and likely represents a small layer of lipids finely distributed on the surface.

In general, the manually inspected mass spectra from various regions appeared to be somewhat similar. They contain the same elements with varying concentrations–Si, CHNOPS, and polyatomic molecules of similar composition. This observation makes it difficult to manually define compounds observed from the Gunflint sample since they seem to represent continually mixing variants. The borders between chemical classes are fused into each other. Thus, the deterministic classification solely based on isotope intensity maps cannot be made. However, we can further explore the chemical variations within different parts of the sample. For example, the spectral features from the top-right corner also show very close proximity to the chemical composition of the host mineral - Si, O, and various Si oxide chains indicate that ablation craters were deep enough to pass through the layer of organic contamination and probe the chemical composition of the original underlying mineral. Lower parts of the isotope maps, shown with black regions (Figure 4-left) after a closer investigation of the mass spectra, were proposed to be from quartz, showing previously described simple chemistry–Si, O, and minor amounts of Na, K, Al. The latter elements (Na, K, Al) could be found as impurities within the chert since they are relatively common in the seawater and could have precipitated together with Si during the time of the rock formation, or they could be from phyllosilicates (clay minerals) that can occasionally occur in the matrix of Gunflint Formation stromatolites, e.g., (Lepot et al., 2017). Since the ^12^C and ^1^H maps outline the structure of the bio-lamination surface, previously identified from the optical microscopy, we can investigate the spectra from the lamination site. The spectra from that area can be characterized by the presence of the bio-relevant elements–with increased concentrations of CHNOPS and an additional minor contribution from Fe, Mn, and Cr. Another notable observation is that spectra from the lamination surface reveal relatively strong polyatomic molecules formation patterns. Various hydrocarbon molecules accompanied by Si oxide chains populate the mass spectra up to 200 m/z.

A dimensionality reduction algorithm was applied over the full mass range of the fs-LIMS imaging data (1–260 amu) to find similar spectra in the dataset. We used the UMAP algorithm (McInnes et al., 2018) to analyze our observations. The first six UMAP components were retrieved from the dataset precompressed with PCA. The UMAP scores were calculated using the Euclidean distance as a metric; every 15 nearest neighbors were used in the construction of the k-nearest neighborhood graph, with a small minimal distance (0.1), and iterated over 400 epochs. This particular set of hyperparameters were found to be appropriate for an approximation of the global structure of the manifold. In the right panel of Figure 4, a distribution of the first three UMAP components is shown. The spectral neighborhood appears to be relatively busy (see point cloud data). However, three main protrusions can be observed from the equal density surface of the UMAP scores. The composition of protruding clusters matches our previous interpretation of the data. The lower part of the plot represents a relatively large cluster of mass spectra acquired from the Quartz-filled matrix. A smaller cluster observed in the vicinity of the main body corresponds to the spectra measured from the area with signatures of organic contamination. It is noteworthy that the contamination cluster is more connected to the main quartz cluster and that the structure of the density surface indicates a smooth transition from pure quartz to the spectra from the surface contamination. The transition structure forms a narrow neck where the similarity of spectra gradually changes from one class to another. From the point cloud data, we could *see* that the contamination cluster is relatively fuzzy, and the fine structure of the transition could be observed on the isodensity surface.

Through the same transition pathway, a cluster of spectra that corresponds to the microfossils preserved within the bio-lamination layer could be observed. In comparison to the cluster of spectra with organic contamination, the density surface of the microfossils cluster forms a separate transition line. The cluster of microfossils forms a smooth identifiable shape, which gradually rises further apart from the quartz and contamination clusters. As one can see, the relative proximity of the spectra located closer to the transition “neck” indicates the ablation of small parts of microfossils. From the investigation of the individual spectra (*see* Figure 3), we have noted that almost all spectra from microfossils contain spectral features from the filling quartz mineral. This observation could be explained by the fact that bodies of microfossils represent partially collapsed and degraded cell walls. The thicknesses of the partially decayed cell walls vary from the first tens of nm to the first hundreds of nm, and these walls are all entombed in the silica matrix. By ablation of small portions of the microfossils and larger portions of the silica matrix, we can explain the smooth transition structure, where similarity of spectra transitions from the clean silica matrix. Thus, the end members of the microfossil cluster represent the best volumetric sampling of microfossils, as well as the best chemical composition of the fossils.

Overall, the volumetric density estimate of the UMAP scores provides a good overview of the spectral types and their transition structures. Also, it is possible to identify outliers (e.g., microscopic inclusions of other minerals) from this graph, for example, by recalculating the isolation forest scores (or any other outlier detection algorithm) – the data points that are weakly connected to the main clusters will have high values, thus, easily identifiable. In the fs-LIMS analysis, where fine chemistry is often of great interest, such information might be valuable because it allows the identification of detached spectra from the bulk of very similar ones.

Figure 4. Left panel–Exemplary isotope intensity maps (warmer colors indicate high concentrations) retrieved from the fs-LIMS mass spectra. The bio-lamination surface (aggregation of microfossils) could be identified from ^12^C and ^1^H maps (bright yellow to red areas), distribution of ^39^K indicates the presence of the surface contamination (upper left corner, bright yellow to the red area). Dark areas on the isotope maps indicate low-intensity regions and correspond to the quartz matrix. To compare with an optical image, *see*Figure 2A. Right panel–Low dimensional structure of the imaging data cube revealed by UMAP. Triangulated mesh represents volumetric isodensity surface of UMAP scores calculated from the 18,000 fs-LIMS mass spectra. Three separate entities could be observed from the spectral neighborhood, namely quartz, contamination, and microfossils. The point cloud data plotted along with the density surface.

The UMAP isodensity estimate reveals the continuous structure of spectral similarities, and therefore it is not clear where to define a boundary between different classes. A density-based clustering approach was used to define discreet classes from the low dimensional UMAP scores. The six UMAP components were used to discretize distributions using a Hierarchical Density-Based Spatial Clustering (HDBSCAN) algorithm (Campello et al., 2013; McInnes et al., 2017). An HDBSCAN provides relatively conservative class assignments compared to other clustering algorithms and potentially more accurate in its predictions. An advantageous side of HDBSCAN over DBSCAN, for example, is that it can find clusters with varying densities, which is precisely the case with our data, where we have an oversampled data from the silicified matrix and a relatively small number of spectra from the microfossils. Moreover, it is possible to calculate the confidence probabilities of the assignment of each spectrum to the cluster, which makes troubleshooting of clustering results more intuitive and less bothersome. However, the downside of the conservative clustering is that some portions of the data might be classified as noise if they do not tightly belong to the densely packed cluster. In contrast to the previous interpretation of UMAP scores, the clustering algorithm found two microfossil populations, a cluster of surface contamination, and quartz from the matrix. The additional cluster of microfossils was hidden on the backside of the quartz-related spectra (*see* Figure 4). The Mapper networks were applied to the spectra registered from the microfossils to visualize the proximity structure between these two classes.

**FIGURE 5:**
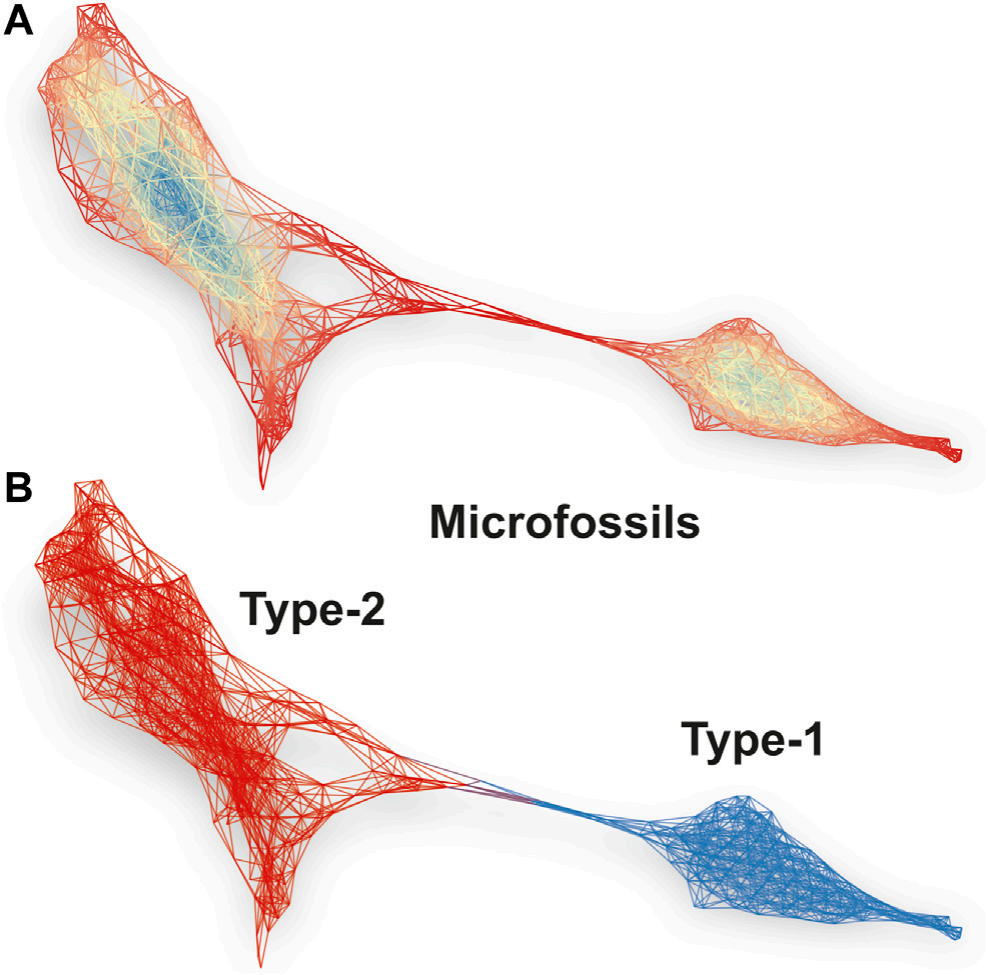
**(A)** Spectral similarity network constructed from 1964 LIMS imaging spectra registered from the microfossils. Each node represents a single or a group of spectra with a significant similarity of intensity profiles. The edges connected with nodes indicate that nodes have one or more shared spectra. The network is colored according to the eigenvector centrality of nodes. A density-based clustering and first three UMAP components were used as a lens to project the data using the Mapper algorithm. The proximity of nodes in the network identifies groups of microfossils and transition structure between two classes. **(B)** The Louvain clustering of the spectral similarity network. The blue part of the network identifies type-1 microfossils, and the red part of the network illustrates spectra registered from the type-2 microfossils. *See* the text for more details.

Figure 5A shows a spectral similarity network constructed from the fs-LIMS spectra registered from the microfossils, using the first three UMAP components as a lens. A python implementation - Kepler Mapper (Van Veen et al., 2019) of the Mapper algorithm (Singh et al., 2007) was used to calculate the similarity network of LIMS spectra. However, other open-source implementations exist - e.g., recently published Giotto-TDA (Tauzin et al., 2020). The density-based clustering was applied to identify clusters within overlapping filter function windows. In total, twenty windows were applied to construct the network with 40% overlap over three UMAP components, forming 8,000 sampling windows and resulting in a complex network with 417 nodes and 2,967 edges (from 1,964 composite spectra registered from the microfossils). Note that the number of filter dimensions is user-defined, and in principle, they might be defined as an *n*-dimensional hypercube, though two-dimensional filters provide the best interpretability. The nodes present in the network indicate groups of fs-LIMS spectra with a high degree of similarity. The nodes might contain one or hundreds of spectra, depending on the size of the filter function window. The edge between nodes is drawn if nodes share the same observations (it might be one or many more spectra). The coloring of the network is conducted according to the eigenvector centralities of the nodes. Blue parts of the network indicate the central nodes, and red parts indicate less connected network components.

The structure of the network identifies the presence of two connected communities. Figure 5B shows the same spectral similarity network as in Figure 5A but colored according to the Louvain modularity, calculated from the network topology. The red part of the network (nodes are not shown) categorizes the spectra identified from the type-2 microfossils, and the blue network indicates the type-1 microfossils. The type-2 microfossils can be characterized by increased proximity to the cluster of spectra registered from the quartz. In contrast to the spectra from type-2, type-1 microfossils are almost completely detached from other groups and form a community of highly connected nodes and correspond to the spectra in a linear protrusion in Figure 4 (right panel). Note that the HDBSCAN and Louvain clustering provides mutually supportive clustering results, although the Mapper networks provide better tolerance to noise, thus allowing for improved clustering performance. In order to check that cluster assignments are not artifactual, we performed a clustering robustness analysis. The Rand Index (RI) metric was used to assess the clustering similarity between 10 random subsamples of the data registered from microfossils. In total, 75% of the data was used to generate random subsamples. The output UMAP subsamples were clustered using the Louvain community detection algorithm. The RI similarity matrix for Louvain clustering of random samples could be found in the supplementary information (*see* Supplementary Table S1 and Supplementary Figure S3). Overall, 45 different clustering pairs revealed an average RI score of 92.5% with a standard deviation of 2%, which indicates that communities shown in Figure 5 are not artefactual and that the cluster assignments are robust. Most of the clustering uncertainty can be attributed to the transition zone between two types of microfossils. The type-2 microfossils reveal more inhomogeneity (*see* Supplementary Figures S1, S2) in comparison to the type-1 microfossils and represent more intermixed with the host mineral material.

The spectral similarity network calculated from the first three UMAP components reveals a better visualization of internal structure and detects outliers and irregularities. Moreover, the force-directed layout (ForceAtlas2 (Jacomy et al., 2014)), applied to the network, exaggerates the positioning of weakly connected nodes, which makes them easier to detect. Moreover, interpretation of the low-dimensional embedding of fs-LIMS data can be easily achieved by coloring the network with original isotope intensities and synthetic features such as isotope ratios. Any other functions might be applied to the data (e.g., Kernel Density Estimate (KDE), Singular Value Decomposition (SVD), and Principal Component Analysis (PCA)), which makes Mapper networks a versatile and powerful tool for insight extraction and hypothesis generation. Furthermore, by reducing the large fs-LIMS intensity space down to a network, we can additionally define a multitude of secondary statistics that could be calculated from the graph topology. Metrics such as centrality, modularity (e.g., *see* Figures 5A,B), average degree, path length (e.g., between the host mineral and microfossils), and many more, can be applied to the specific minerals and microfossils to define the multiparametric space further and enhance the potential for definitive identification.

Figure 5A. Spectral similarity network constructed from 1,964 LIMS imaging spectra registered from the microfossils. Each node represents a single or a group of spectra with a significant similarity of intensity profiles. The edges connected with nodes indicate that nodes have one or more shared spectra. The network is colored according to the eigenvector centrality of nodes. A density-based clustering and first three UMAP components were used as a lens to project the data using the Mapper algorithm. The proximity of nodes in the network identifies groups of microfossils and transition structure between two classes. Figure 5B. The Louvain clustering of the spectral similarity network. The blue part of the network identifies type-1 microfossils, and the red part of the network illustrates spectra registered from the type-2 microfossils. See the text for more details.

**FIGURE 6:**
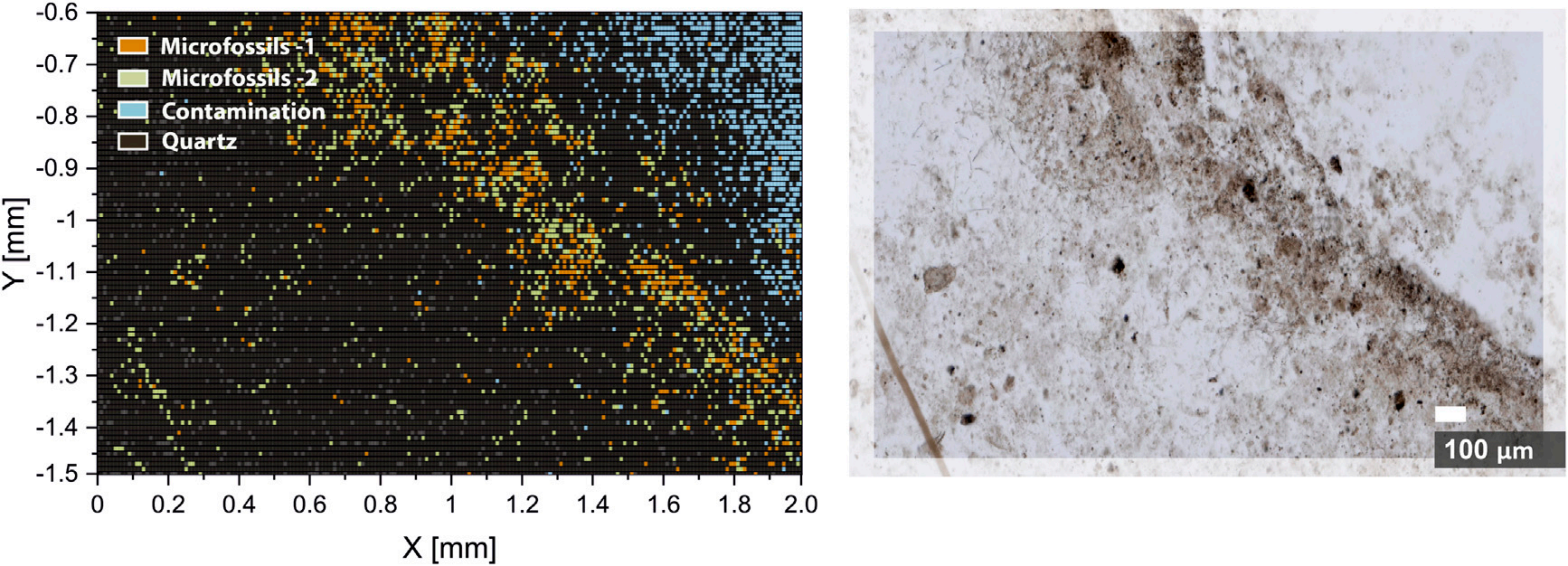
Hierarchical density-based spatial clustering (HDBSCAN) of six UMAP components of the imaging dataset (left panel). The orange pixels represent spectra registered from the type-1 microfossils. The green pixels represent spectra registered from the type-2 microfossils. The blue pixels represent spectra registered from the surface contamination. Black and grey pixels–spectra registered from the quartz matrix of the Gunflint chert. Right panel–the optical microscopy image of the analyzed area. Note the aligned distribution of classified spectra with the bio-lamination surface crossing the image.

The overall results of the density-based clustering can be seen in Figure 6. Clustering results reveal a very close match with results of optical microscopy (*see* Figure 6, right panel) and conclusions from previous single isotope maps investigations. Moreover, we have identified two types of microfossils and a contamination zone, which were not acknowledged from the microscope image. The type–1 microfossils represent spectra obtained from the microfossils with the best microfossil over host (matrix mineral) sampling ratio. Thus, spectra from type-1 can be counted as the most representative of microfossils. On the other hand, type-2 represents the microfossils with an increased contribution from the host mineral, which is also shown in Figure 6. The chemical composition of type-1 microfossils can be characterized with increased content of carbon and oxygen (^12^C, ^12^C^2+^, and ^16^O^2+^ peaks in the mass spectra), whereas type-2 microfossils contain less ^12^C and more hydrocarbons, which indicates lower volumetric ablation and colder plasma temperatures, thus, more prevalent recombination processes. Higher plasma temperatures observed in the type-1 microfossils can be attributed to the higher volumetric contribution from absorptive kerogen. This observation also finds confirmation from the spatial distribution of microfossils. In Figure 6, the first type is mainly distributed in the densely populated area (*see* Figure 6, right panel), in contrast to type-2, which is largely distributed outside of the dense zone, and more likely to be sampled with larger portions of the host mineral. The identification of microfossils from the host mineral using fs-LIMS and low dimensional analysis provides topological biosignatures. As it was shown in Figures 4, 5, the structure of spectral similarities identifies the positionings of spectra from different classes and provides means for identification, classification of large datasets, and has a potential for the prediction of spectral classes from previously unseen spectra, given that a sufficiently rich spectral library is provided.

Figure 6. Hierarchical density-based spatial clustering (HDBSCAN) of six UMAP components of the imaging dataset (left panel). The orange pixels represent spectra registered from the type-1 microfossils. The green pixels represent spectra registered from the type-2 microfossils. The blue pixels represent spectra registered from the surface contamination. Black and grey pixels–spectra registered from the quartz matrix of the Gunflint chert. Right panel–the optical microscopy image of the analyzed area. Note the aligned distribution of classified spectra with the bio-lamination surface crossing the image.

## Discussion

The identification and chemical characterization of minerals and prospective biosignatures from large spectral databases generated using fs-LIMS as well as other *in-situ* spectroscopic techniques is a longstanding problem that can be generalized to other analytical methods as well. For example, other important methods proposed for *in-situ* space exploration, such as Laser-Induced Breakdown Spectroscopy (LIBS) (e.g., ChemCam, currently operates on Mars as part of the Mars Science Laboratory), Raman spectroscopy (i.e., Raman Laser Spectrometer (RLS), one of the Pasteur Payload instruments from ExoMars), and a large variety of other techniques rely on harvesting large spectral information from the analyte material. This spectral information is often hard to interpret due to the large dimensionality, complexity, and size of generated datasets. Outside of the context of space exploration, in the field of analytical chemistry, similar data analytical challenges are often encountered in the laboratory. For example, Secondary Ions Mass Spectrometry (SIMS) or Liquid Chromatography Mass Spectrometry (LC-MS), as high-throughput techniques, provide hundreds of mass lines per spectrum, and the output spectral dataset is not always easy to interpret. As was shown in this contribution, analysis of fs-LIMS data using topological methods reveals a fast and accurate description of spectral classes and provides a good understanding of transitional structures. In the low dimensional domain, it might be easier to generate insights and formulate a hypothesis, thus accelerating the extraction of knowledge from the given sample.

The analysis of data generated by using our fs-LIMS system might also be of use for future investigations of Precambrian rocks containing signatures of putative microfossils. The Gunflint sample is rare amongst Precambrian rocks as it exhibits an exceptional level of morphological and chemical preservation, so there is little argument over the biogenicity of the encased organic material (Barghoorn and Tyler 1965; Lepot et al., 2017; Wacey et al., 2012). However, traces of early life can be destroyed or heavily altered by heat, pressure, and time (diagenetic alteration and later metamorphism). As was briefly discussed before, the full mass range spectral proximity analysis provides a means for the classification of chemically similar entities. For example, organic contamination and microfossils - similar compounds (both contain CHNOPS and Si mass peaks), can be distinguished using topological methods (*see* Figures 4–6). A big challenge in the field of Precambrian micropaleontology surrounds the fact that altered and reduced carbon found in ancient rocks could potentially be of biological origin but could also have been created by abiotic processes. For example, Fischer-Tropsch type synthesis might be responsible for the presence of some abiotic hydrocarbons in Precambrian formations (Brasier et al., 2002). However, we speculate that synthetic products of Fischer-Tropsch-like reactions will have a distinct spectral profile (e.g., polyatomic plasma chemistry products might be different), and therefore corresponding topological positioning is expected to be distinguishable from *bona fide* microfossils. Thus, there is a hope that signs of life in controversial samples might be successfully identified using sensitive methods and full-feature-based topological representations.

The current state of space exploration also faces similar challenges in the field of *in-situ* chemical analysis of solids on planetary bodies. For example, the ns-LIMS instrument proposed for Europa (Ligterink et al., 2020) reported the identification of extremely low quantities of biological and abiotic amino acids from well-defined extracts at the fmole level. However, more complex molecules (e.g., proteins, polysaccharides, etc.) combined with various undefined matrices will likely form complex fragmentation patterns with hundreds of significant mass lines, thus, making the identification challenging. The topological representation, in this case, might provide a number of compounds present in the measured mixture and their similarity to the predefined classes.

The unsupervised identification of minerals from fs-LIMS chemical imaging datasets might also be of use in the determination of relative sensitivity coefficients (RSC’s). The fs-LIMS is a quantitative method; however, it requires the establishment of RSC’s, which are matrix dependent. With an introduction of fs-LIMS, some matrices have been reduced to unity (RSC = 1, no correction needed). However, non-absorptive samples such as glasses typically still require the determination of RSC’s for quantitative measurements. In the case of exploratory analysis, where we do not know the sample (i.e., field exploration of Martian samples), if one would know the stoichiometry of the investigated mineral, it is possible to recalculate correction factors for major elements, and then through RSC’s dependence on atomic orbital ionization energy recalculate concentrations of minor and trace elements (Tulej et al., 2021). The key component here is the identification of the mineral, and as we described above, topological methods provide a means to do that.

Here we also need to point out several caveats regarding the analysis of fs-LIMS data. First, at multiple stages, the data analysis procedures require a set of hyperparameters to be chosen. For example, in UMAP embedding and Mapper network construction, we used Euclidean and cosine distances, respectively, and defined the number of neighbors, number of clusters, and filter functions. However, a more rigorous study of the effect of hyperparameters needs to be assessed in future studies regarding the analysis of fs-LIMS data or data generated by other spectroscopic techniques. Nevertheless, a recent contribution by (Belchı et al., 2020) provides an insight into the numerical stability of Mapper-type algorithms. It was shown that reliable Mapper output could be identified as a local minimum of instability, regarded as a function of Mapper input parameters. Other statistical solutions were proposed to circumvent testing large parametric spaces and keep the most representative Mapper settings (Carriere et al., 2018). Furthermore, we have used UMAP scores as a lens in the construction of the similarity network; however, a large variety of other functions might be used, and their impact on visualizations needs to be assessed. It would also be valuable to implement into the analysis pipeline some domain-specific lenses for technical usage (i.e., mass resolution, mass accuracy, etc.), which will improve the extraction of quality metrics.

Overall, in addition to the account of topological descriptors of early life, we hope that our analysis will facilitate, in time, a predictive approach in the field of study of early life. The approach described here might be expanded to more powerful, state-of-the-art standalone laboratory instrumentation (e.g., high-resolution LIMS, SIMS, LA-ICP-MS), where data quality might provide a whole new quantification perspective.

## Conclusion

Our contribution offers several important conclusions for *in-situ* space research. First, the miniature fs-LIMS system combined with topology-based data analysis demonstrates the utility and sensitivity to distinguish organically preserved microfossils from organic contamination and inorganic host mineralogy. Second, the proposed approach might be extended to other complex samples with multimineral compositions and used with other high-resolution spectrometric or spectroscopic methods. Third, our approach - full spectral mass range convolution down to a similarity network for life detection stands out from multielement methods. It offers great flexibility and could be further expanded to study the chemical discrepancies between individual populations of microfossils. Furthermore, our analysis reveals fine transition structures between classes and the detection of outliers. Last, the fs-LIMS system, in combination with topological methods, enables faster data analysis, accelerates the formulation of hypotheses, and the generation of insights for mineralogical compositions of investigated samples.

## Data Availability

The original contributions presented in the study are included in the Supplementary Materials, further inquiries can be directed to the corresponding author.
