# Supplementary material for: On Topological Analysis of fs-LIMS Data. Implications for in Situ Planetary Mass Spectrometry
Source: Front Artif Intell. 2021 Aug 23;4:668163. doi: 10.3389/frai.2021.668163 (PMC8419467; doi:10.3389/frai.2021.668163)
Supplement: Supplementary file 2 [file DataSheet6.PDF]

Figure S1. The Mapper networks constructed from subsampled data, shaped (1473, 6) and using lens shaped (1473, 3) - 75% of original data
Number of filter cubes = 20, percent of overlap = 0.5
UMAP projection dimensions - 0, 1, 2
Clusterer - DBSCAN (epsilon = 25, minimal number of samples = 8, metric = 'cosine')

Subsample-1

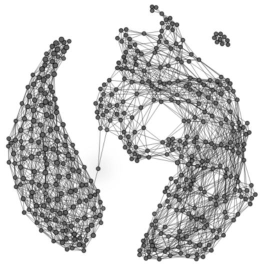

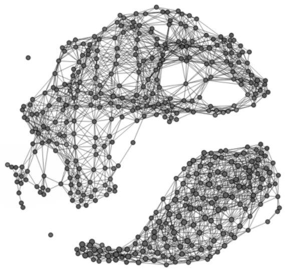

Subsample-6

Subsample-2

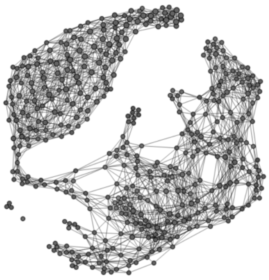

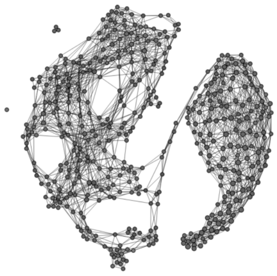

Subsample-7

Subsample-3

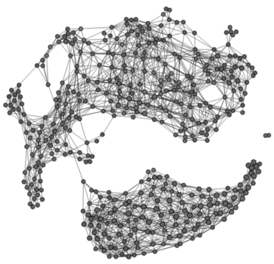

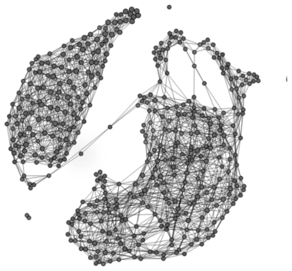

Subsample-8

Subsample-4

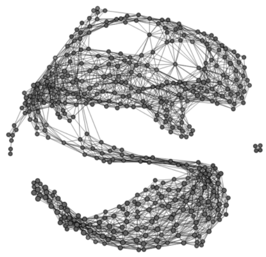

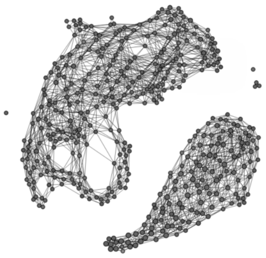

Subsample-9

Subsample-5

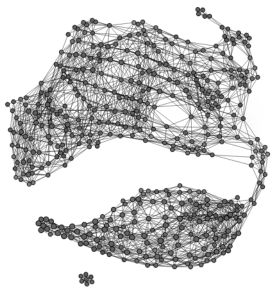

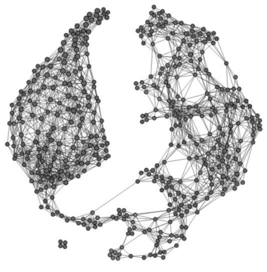

Subsample-10

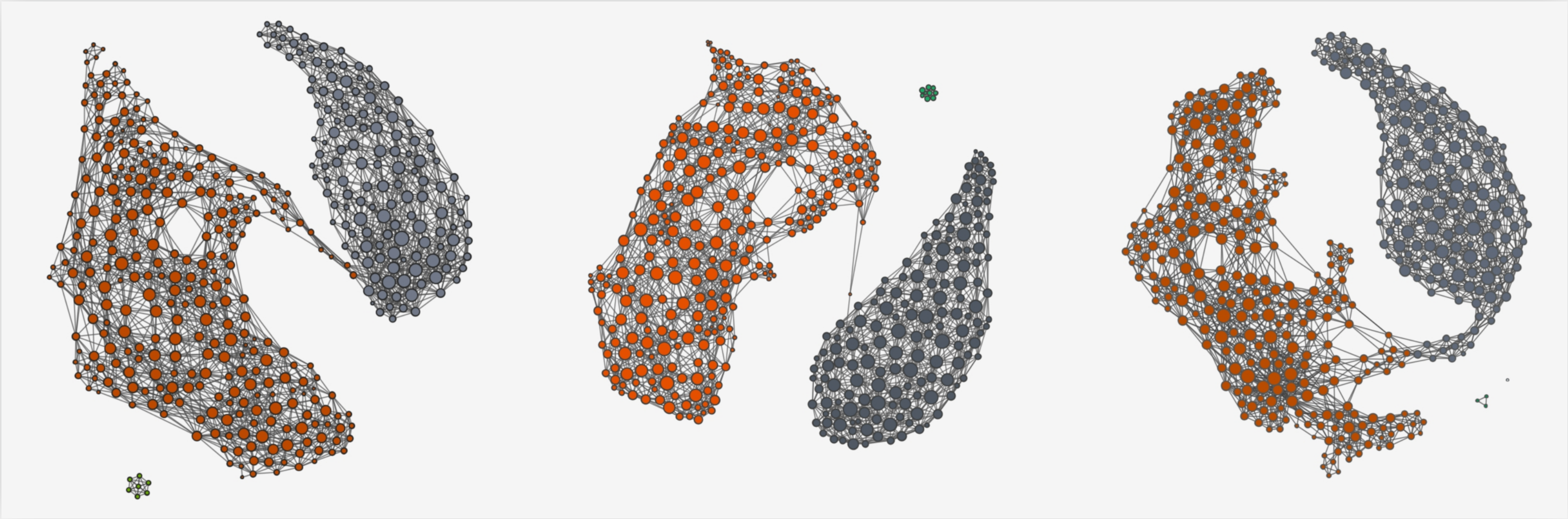

Figure S2. Exemplary Louvain clustering of random subsamples.
The type-1 microfossils (grey nodes) reveal stable clustering results. Whereas, type-2 microfossils (red nodes) show an increased level of inhomogeneity.
Node sizes are scaled according to the nodal degree.

Rand Index

Samples	Subsample1	Subsample2	Subsample3	Subsample4	Subsample5	Subsample6	Subsample7	Subsample8	Subsample9	Subsample10
Subsample1	1.000	0.941	0.920	0.957	0.909	0.956	0.934	0.942	0.938	0.955
Subsample2	0.941	1.000	0.902	0.934	0.918	0.943	0.933	0.906	0.920	0.919
Subsample3	0.920	0.902	1.000	0.926	0.871	0.923	0.892	0.929	0.913	0.926
Subsample4	0.957	0.934	0.926	1.000	0.901	0.932	0.937	0.959	0.935	0.972
Subsample5	0.909	0.918	0.871	0.901	1.000	0.913	0.910	0.875	0.907	0.907
Subsample6	0.956	0.943	0.923	0.932	0.913	1.000	0.920	0.912	0.939	0.925
Subsample7	0.934	0.933	0.892	0.937	0.910	0.920	1.000	0.924	0.920	0.926
Subsample8	0.942	0.906	0.929	0.959	0.875	0.912	0.924	1.000	0.913	0.966
Subsample9	0.938	0.920	0.913	0.935	0.907	0.939	0.920	0.913	1.000	0.926
Subsample10	0.955	0.919	0.926	0.972	0.907	0.925	0.926	0.966	0.926	1.000

Table S1. Rand Index (RI) scores calculated for Louvain clustering of random subsamples (1473 mass spectra or 75% of original data). Minimal number of samples for DBSCAN clusterer were set to 1 to avoid unequal sampling. Majority of the RI values are above 0.9.

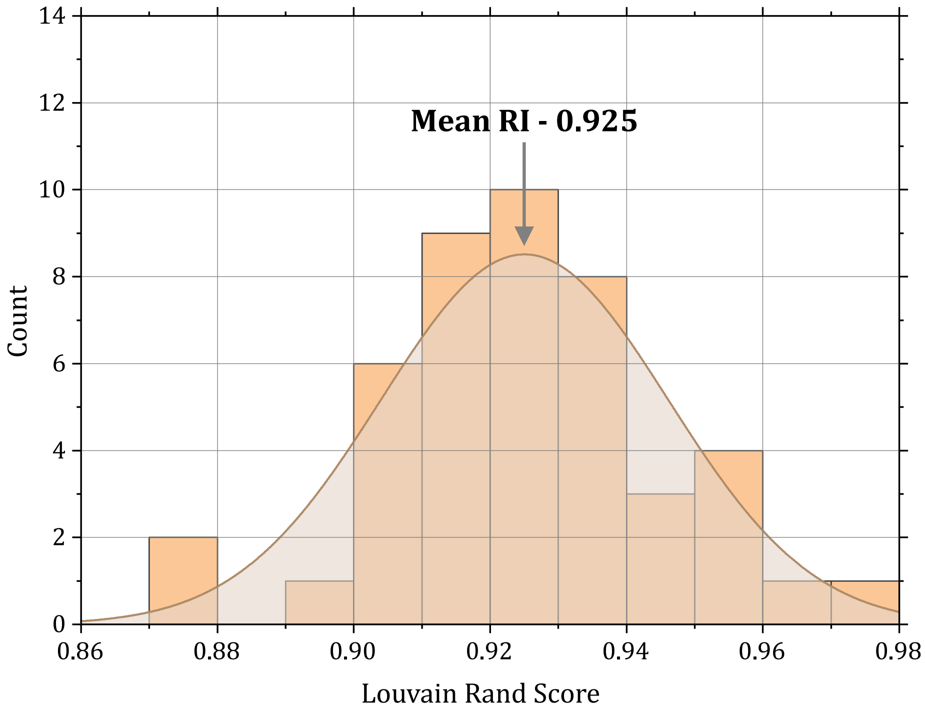

Figure S3. Distribution of the RI scores.
Mean value equals to 0.925 (derived from 45 observations). Standard deviation - 0,02. Maximum value - 0.97; Minimum value - 0.87. Distribution indicates robust clustering results.
